# High Performance p-i-n Photodetectors on Ge-on-Insulator Platform

**DOI:** 10.3390/nano11051125

**Published:** 2021-04-27

**Authors:** Xuewei Zhao, Guilei Wang, Hongxiao Lin, Yong Du, Xue Luo, Zhenzhen Kong, Jiale Su, Junjie Li, Wenjuan Xiong, Yuanhao Miao, Haiou Li, Guoping Guo, Henry H. Radamson

**Affiliations:** 1 CAS Key Laboratory of Quantum Information, University of Science and Technology of China, Hefei 230026, China; zhaoxuewei@ime.ac.cn (X.Z.); haiouli@ustc.edu.cn (H.L.); gpguo@ustc.edu.cn (G.G.); 2 Key Laboratory of Microelectronics Devices & Integrated Technology, Institute of Microelectronics, Chinese Academy of Sciences, Beijing 100029, China; linhongxiao@ime.ac.cn (H.L.); duyong@ime.ac.cn (Y.D.); kongzhenzhen@ime.ac.cn (Z.K.); sujiale@ime.ac.cn (J.S.); lijunjie@ime.ac.cn (J.L.); xiongwenjuan@ime.ac.cn (W.X.); 3 Institute of Microelectronics, University of Chinese Academy of Sciences, Beijing 100049, China; 4 Research and Development Center of Optoelectronic Hybrid IC, Guangdong Greater Bay Area Institute of Integrated Circuit and System, Guangzhou 510535, China; luoxue@giics.com.cn; 5 Department of Electronics Design, Mid Sweden University, Holmgatan 10, 85170 Sundsvall, Sweden

**Keywords:** GOI, photodetectors, dark current, responsivity

## Abstract

In this article, we demonstrated novel methods to improve the performance of p-i-n photodetectors (PDs) on a germanium-on-insulator (GOI). For GOI photodetectors with a mesa diameter of 10 μm, the dark current at −1 V is 2.5 nA, which is 2.6-fold lower than that of the Ge PD processed on Si substrates. This improvement in dark current is due to the careful removal of the defected Ge layer, which is formed with the initial growth of Ge on Si. The bulk leakage current density and surface leakage density of the GOI detector at −1 V are as low as 1.79 mA/cm^2^ and 0.34 μA/cm, respectively. GOI photodetectors with responsivity of 0.5 and 0.9 A/W at 1550 and 1310 nm wavelength are demonstrated. The optical performance of the GOI photodetector could be remarkably improved by integrating a tetraethylorthosilicate (TEOS) layer on the oxide side due to the better optical confinement and resonant cavity effect. These PDs with high performances and full compatibility with Si CMOS processes are attractive for applications in both telecommunications and monolithic optoelectronics integration on the same chip.

## 1. Introduction

Silicon complementary metal oxide semiconductor (CMOS) technology could be used to integrate optical data communication and electrical data computing to achieve electron–photon synergy. Combining the advantages of photons and electrons can overcome some of the bottlenecks which microelectronics technology encounters with the development of Moore’s Law, and open more directions for the continuation of Moore’s Law [1,2]. Photodetectors (PDs) are the core devices of high-performance electro-optic conversion. Si photodetectors (PDs) are not attractive in optical communication because the cut-off wavelength of their absorption spectrum is 1100 nm. Ge has emerged as a leading contender for optoelectronic devices due to its pseudo-direct bandgap behavior, large absorption coefficient in the near-infrared region, lower cost, and compatibility with CMOS processing compared to III–V materials. This makes Ge PDs very promising in optoelectronic applications.

There are still some practical difficulties to overcome in the research of Ge PDs, especially for the Ge epitaxy on Si. People first attempted to epitaxially grow Ge on Si with a low dislocation density, mainly because the mobility of electrons and holes in Ge is higher than that in Si [3]. For high-quality Ge epitaxy on Si, the greatest challenge is the 4.2% lattice mismatch ratio between these two elements. This mismatch will result in high surface roughness after growth and high threading dislocation densities in the Ge epitaxial layer, which will hinder the integration of Si and Ge devices and affect device epitaxy on Si [4,5,6]. SiGe buffer layers were used to reduce the number of threading dislocations in the Ge layer. By carefully adjusting the composition of the SiGe layers, many threading dislocations can be bent or terminated at the heterojunction interfaces, which greatly reduces the dislocation density in the Ge layer [7,8]. A two-step growth method has also been proposed, which can effectively prevent island-like growth, and subsequent annealing can greatly reduce the linear dislocation density [9,10,11]. Many efforts have been taken to improve the quality of the Ge layer on the Si substrate in order to improve the performance of Ge normal-incident PDs [12], waveguide PDs (WGPDs) [13,14], metal–semiconductor–metal PDs (MSM PDs) [15], etc. However, the fundamental problem of high defective regions at the epitaxial Ge/Si interface has not yet been solved. Various methods have been widely reported to optimize the process of Ge. Compared to the Ge-on-Si structure, the quality of the Ge layer on Ge-on-insulator (GOI) platforms can be significantly improved. During the preparation of GOI substrates, the low-temperature Ge layer with high defect density was removed, resulting in fewer generation/recombination centers in the Ge crystal [16]. In ref [17], the leakage current of Ge p-i-n photodiodes on a GOI substrate with threading dislocation density (TDD) of ~3.2 × 10^6^ cm^−2^ was reduced by 53-fold from one with a TDD of ~5.2 × 10^8^ cm^−2^. In addition, the introduction of an insulator layer between the Si and Ge can provide better optical confinement for the Ge active layer, enhancing the optical responses of the devices [18]. The direct absorption edge of Ge at 1550 nm limits the application of Ge PD in the C-band (1530–1560 nm) and L-band (1560–1625 nm). In order to achieve high photodetection efficiency in the Ge layer, the optical absorption coefficient must be enhanced by narrowing the direct bandgap energy. An effective approach to narrow the bandgap is to apply enhanced tensile lattice strain. Tremendous efforts have been made to enhance tensile strain in Ge, including the introduction of GeSn [19,20], microbridge structures [21,22], external stressors of SiN_x_ [23,24], etc. To date, only a few Ge PD photoelectric detection ranges have been extended due to tensile strain on GOI substrates [25,26,27,28,29], and to the best of our knowledge, there is no report on the performance comparison of p-i-n detectors prepared on GOI with different oxide thickness.

In this work, we propose novel methods to improve the p-i-n photodetectors on the GOI substrate. Compared to the PD with low-temperature Ge on the Si substrate, the responsivity of the GOI detector is remarkably improved from 0.32 to 0.5 A/W under 1550 nm wavelength, and from 0.54 to 0.9 A/W under 1310 nm wavelength, increases of 56% and 67%, respectively. The measurement results show that the GOI PDs have lowest dark current of 2.5 nA because of better crystal quality. We also found that the optical performance of the GOI PD could be remarkably improved by integrating a tetraethylorthosilicate (TEOS) layer on the oxide side due to the better optical confinement and resonant cavity effect. We have realized the fabrication and experimental verification of ultra-low dark current and high responsivity GOI photodetectors, for which the detection range can be extended to 1630 nm. This method provides a good foundation for the realization of single-chip optoelectronic integration on large wafers in the future. 

## 2. Materials and Methods

In this study, the Ge layers were deposited on p-type Si (001) 200 mm wafers in a reduced pressure chemical vapor deposition (RPCVD) reactor (ASM Epsilon 2000, Almere, The Netherlands). Ge epitaxy (epi-Ge) was performed in two steps, at 400 °C (low temperature or LT) and 650 °C (high temperature or HT), followed by an annealing of 820 °C for 10 min. The TDD was estimated to be 2.79 × 10^7^ cm^−2^ in the Ge layer. The top 700 nm Ge layer was boron-doped to form P^+^-Ge. The growth parameters were carefully optimized to avoid dopant precipitates in the Ge layer [30]. At the same time, a thermal oxide layer of 523 nm was formed on the handle silicon wafer. Later, a 10 nm Al_2_O_3_ layer was deposited on the epi-Ge wafer to increase the adhesion for bonding. At this stage, two groups of samples were prepared for bonding; one group was directly bonded to the oxide wafer, but the other one with the TEOS layer was thinned to ~300 nm by chemical mechanical polishing (CMP). Then, the Si of the bonded wafer was removed through a combination of mechanical grinding and wet etching in tetramethylammonium hydroxide (TMAH) solution to form the GOI wafer. Afterwards, a CMP process was applied to remove the defected LT-Ge layer on the top. In these GOI wafers, the final Ge thickness was ~2 µm but the oxide thickness for the sample with no TEOS was 523 nm, whereas for the other one it was ~800 nm. In our experiments, the grown Ge layer on Si was considered as the reference sample.

**Figure 1:**
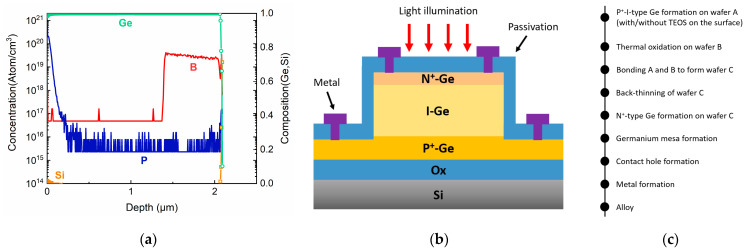
(**a**) SIMS data, (**b**) cross-section schematic, and (**c**) main process flow of GOI p-i-n photodetectors.

Finally, a 100 nm thick top n-type Ge layer was formed by ion implantation twice at a dose of 1 × 10^15^ cm^−2^ and an energy of 18 keV. After each implantation, an annealing treatment at 500 °C in hydrogen for 60 s was performed. The doping level of the n-type layer was estimated as ~2 × 10^20^ cm^−3^. The dopant profile of the PIN structure was evaluated by secondary ion mass spectroscopy (Figure 1a). 

Pixels with diameter sizes of 10, 20, 40, 60, 80, and 100 μm were defined and etched down to p-type Ge (bottom contact), followed by a 300 nm thick SiO_2_ deposition as a passivation layer by plasma-enhanced chemical vapor deposition (PECVD). After contact, electrode holes were formed by dry etching and wet etching, a 10 nm Ni layer was deposited and rapid thermal annealing (RTA)-treated at 450 °C to form NiGe. The NiGe reduced the contact resistivity to 1.3 × 10^−5^ Ω∙cm^2^, which was one of the important factors for low dark current in our detectors. Ni is a better choice compared to Co and Pt, which require higher annealing temperatures which cause dopant diffusion in the detector structure [31,32,33]. Later, a Ti/TiN/AlCu stack with thickness of 50 nm/10 nm/400 nm was deposited and etched to form metal electrodes. A schematic of the processed detector structure is shown in Figure 1b, and the main process flow of the GOI detector is shown in Figure 1c.

**Figure 2:**
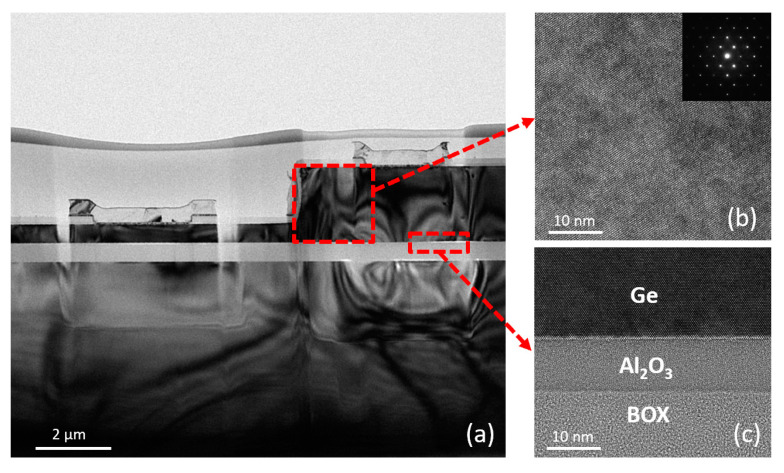
(**a**) Cross-sectional transmission electron microscopy (TEM) image of GOI p-i-n photodetectors. (**b**) High-resolution TEM image with its selected area diffraction (SAD) image (the inset) in the Ge layer, and (**c**) the Ge/Al_2_O_3_/SiO_2_ interface.

Figure 2a displays the cross-sectional transmission electron microscopy (TEM) (Thermo Fisher Talos, Brno, Czech Republic) image of the entire GOI photodetector with the diameter of 100 μm. An enlargement highlights the lattice arrangement of the Ge layer in Figure 2b. The crystalline quality of Ge layer was high and no obvious threading dislocations were observed, because in our process the defected LT-Ge layer was totally removed, and only the high-quality HT-Ge layer remained. The selected area diffraction (SAD) result of the Ge intrinsic layer is shown in the inset of Figure 2b, which has a very regular arrangement of spots, indicating excellent crystal quality. Figure 2c is an enlarged TEM image at the interface, showing a flat interface with no dislocations between the Ge/Al_2_O_3_/SiO_2_ layers. The materials and processes used for device fabrication can be implemented in a standard CMOS process flow.

## 3. Results and Discussions

### 3.1. Dark Current

**Figure 3:**
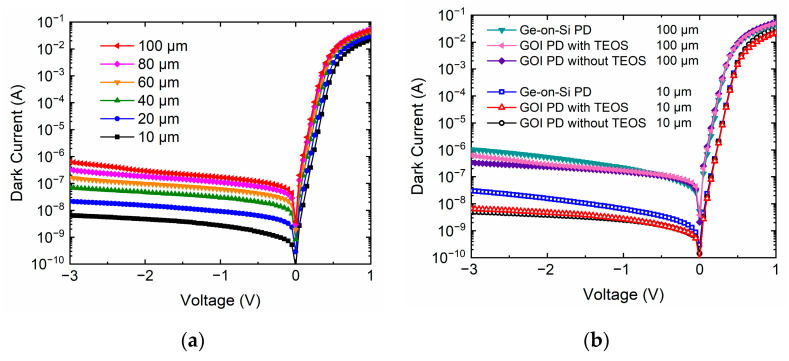
Dark currents of (**a**) GOI detectors with diameters of 10, 20, 40, 60, 80, and 100 μm, and (**b**) detectors on three types of substrates with diameters of 10 and 80 μm.

The p-i-n PDs were characterized with respect to their electrical properties. The dark current of the photodiode is not only an indication of material quality, but also determines optical receiver sensitivity [10]. The dark current–voltage characteristics of the devices on GOI with various mesa radii were measured with a Keithley 4200-SCS semiconductor parameter analyzer (Cleveland, OH, USA) at room temperature, as shown in Figure 3a. The dark current curves exhibited a remarkable rectifying behavior with a high on/off current ratio near 10^8^ between 1 and −1 V. Figure 3b displays the dark current comparison of GOI PDs and Ge-on-Si PDs with diameters of 10 μm and 100 μm. For Ge-on-Si PD with 10 μm diameter, the dark currents are 6.4, 15.6 and 30.1 nA at −1, −2 and −3 V. The detector on GOI with TEOS with diameter of 10 μm exhibited dark currents as low as 2.7, 4.7, and 6.6 nA at −1, −2, and −3 V. The detector on GOI without TEOS with a diameter of 10 μm exhibited dark currents as low as 2.5, 3.8 and 5 nA at −1, −2 and −3 V. The dark currents of GOI PDs without TEOS were 7.8, 27 and 100 nA for diameters of 20, 40, and 80 μm at −1 V, respectively. The defects/dislocations in the Ge layer increased the trap-assisted tunneling (TAT) leakage current, and carrier tunnels through the center of the Shockley–Read–Hall (SRH) under a relatively high reverse bias [34]. The GOI PDs exhibited ultra-low dark current because of the absence of a defective region compared to Ge-on-Si PDs.

The dark current density (*J_total_*) can be divided into the bulk leakage current density (*J_bulk_*) and the surface leakage density (*J_surf_*) using the following equation [6]:

*J_total_* = *J_bulk_* + 4 *J_surf_*/*D* (1)

**Figure 4:**
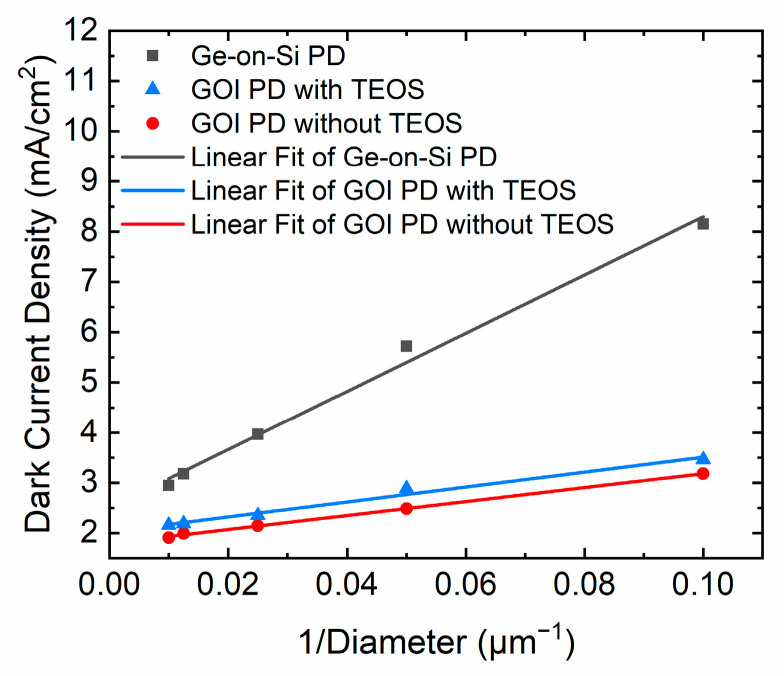
Dark current density (*J_total_*) versus 1/D of PDs at −1 V reverse bias.

Figure 4 displays the total dark current densities (*J_total_*) of different photodetectors at −1 V versus 1/D, where D is the mesa diameter of the device. Table 1 shows the dark current density comparison of Ge-on-Si PDs and GOI PDs. Compared with Ge-on-Si PDs, both surface leakage current and bulk leakage current of GOI PDs have been significantly reduced. The *J_bulk_* and *J_surf_* values of the GOI detector without TEOS extracted from Figure 4 were as low as 1.79 mA/cm^2^ and 0.34 μA/cm, respectively. This low bulk dark current density of 1.79 mA/cm^2^ is one of the lowest reported dark current density values among the Ge p-i-n photodetectors [35,36], which confirms the excellent Ge crystal quality. The *J_surf_* of 0.37 μA/cm indicates excellent surface passivation, resulting in lower surface leakage current. 

### 3.2. Responsivity

**Figure 5:**
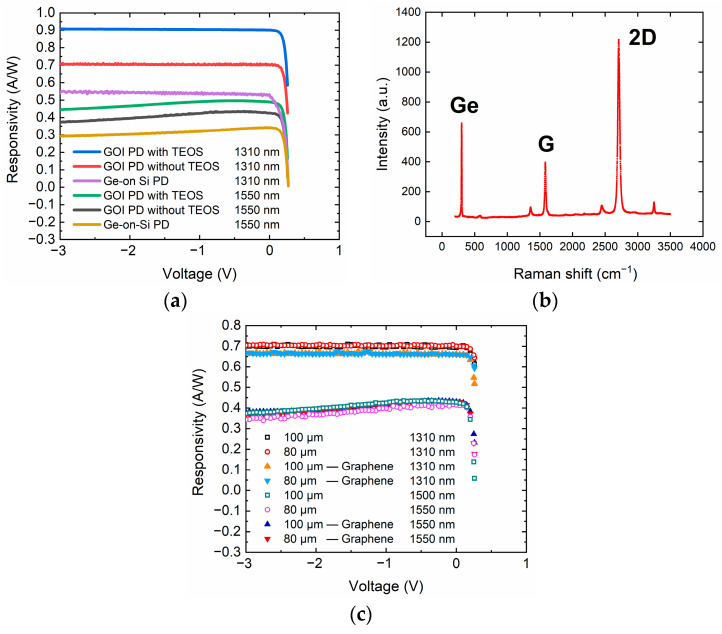
(**a**) Responsivity–voltage characteristics for illuminated PDs with a diameter of 80 μm on three types of detectors. (**b**) Raman spectra of transferred graphene on the detector. (**c**) Responsivity–voltage characteristics for illuminated GOI PDs (without TEOS) with or without graphene on the surface.

The photo currents of GOI photodetectors were measured at room temperature using an Agilent B1500A semiconductor parameter analyzer, a probe station, a laser with a wavelength of 1310 nm, and a tunable laser (1500–1630 nm). The photocurrents of the three types of detectors, which are Ge-on-Si PDs, GOI PDs without TEOS, and GOI PDs with TEOS, were measured, respectively. The thickness of the intrinsic Ge layer of all detectors was almost the same (~1.2 μm). The laser output was measured by a calibrated commercial reference detector. The incident light was coupled into the detectors through a single-mode fiber perpendicular to the surface. The spot-size of the fiber was about 3–5 μm. The light power was verified to be 1 mW by a calibrated commercial reference detector. The responsivity parameter is defined as follows:

*R* = *I_ph_/P_o_* = *ηqλ*/*hc* (2)

where *I_ph_* is the photocurrent, *P_o_* is the optical power incident on the PD, *η* is the quantum efficiency, *q* is the electrical charge, *λ* is incident light wavelength, *h* is Planck’s constant, and *c* is the speed of light. The responsivity characteristics of different photodetectors under wavelength of 1550 and 1310 nm are shown in Figure 5a. The optical responsivity of GOI PD without TEOS at 1550 and 1310 nm was 0.43 and 0.7 A/W at −1 V, corresponding to the external quantum efficiencies of 34.4 and 66.3%, respectively. For GOI PD with TEOS, the responsivity at 1550 and 1310 nm was 0.5 and 0.9 A/W at −1 V, corresponding to the external quantum efficiencies of 40 and 85.2%, respectively. The saturation of the optical responsivity values at 0 V bias revealed that the photodetector configuration allowed a complete photogenerated carrier collection without bias. The responsivities of the GOI PDs with TEOS at 1550 nm were 0.50, 0.47, and 0.45 A/W at −1 V, −2 V, and −3V, respectively. The responsivity at λ = 1310 nm was almost constant throughout the reverse bias region, while the responsivity around the band edge (λ = 1550 nm) slightly decreased with the increasing reverse bias because of the Franz–Keldysh effect (FKE) [37]. 

In order to improve the performance of Ge PD, an absorption graphene layer was placed on the detector’s chip. Raman analysis was performed to confirm the quality of graphene after the transfer process, as shown in Figure 5b. The Ge (≈800 cm^−1^), G (≈1600 cm^−1^) and 2D peaks (≈2700 cm^−1^) with an almost-invisible D peak (≈1300 cm^−1^) on the SiO_2_ indicate that the monolayer graphene was transferred successfully. Figure 5c shows the responsivity characteristics of GOI PDs with or without graphene at 1550 nm and 1310 nm, where 80 and 100 μm are the diameters of the detectors. Although the initial idea behind using graphene was to absorb infrared (IR) radiation, in these measurements, the detector with a graphene layer showed no significant improvements in the responsivity values. 

**Figure 6:**
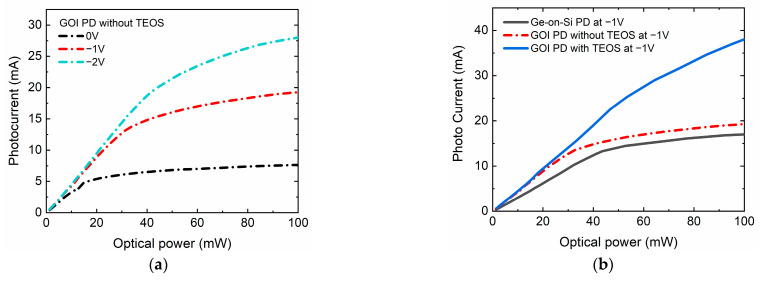
(**a**) Saturated photocurrent curves of the GOI PD without TEOS with 80 μm diameter at bias voltages of 0, −1, and −2 V. (**b**) Saturated photocurrent curves of the normal Ge-on-Si photodetector, GOI PD without TEOS, and GOI PD with TEOS at −1 V.

In order to study the high-power characteristics of photodetectors, we used a semiconductor parameter analyzer, a probe station, a 1550 nm laser, and an erbium-doped fiber amplifier (EDFA) to measure the photocurrent under different optical powers at room temperature. The laser output was amplified by the EDFA and introduced on the top surface of the photodetectors. Figure 6a shows the saturated photocurrent curves of the GOI PDs without TEOS with 80 μm diameter at a bias voltage from 0 to −2 V. The photocurrent gradually increased until it was saturated as the optical power increased. The saturated photocurrent increased with the increase in the bias voltage, because the intensity of electric field became stronger with the increase in the bias voltage and more photogenerated carriers were brought to the electrode, which eventually led to the saturation of the photocurrent [12]. The photocurrents of GOI PDs without TEOS under 100 mW incident light power were 28, 19.3, and 7.6 mA at bias voltages of −2, −1 and 0 V, respectively.

The high-power characteristics of photodetectors on different types of substrates were investigated, as shown in Figure 6b. The black, red, and blue lines represent the saturated photocurrents curves of the Ge-on-Si PD, GOI PD without TEOS, and GOI PD with TEOS with the same diameter of 80 μm, respectively. Compared to the Ge-on-Si PD, the saturated photocurrent of the GOI PD without TEOS was improved from 17 to 19 mA at −1 V. The saturated photocurrent of the GOI PD with TEOS seemed to be higher than 40 mA when the incident light power exceeded 100 mW, which is twice that of the GOI PD without TEOS at −1 V. 

**Figure 7:**
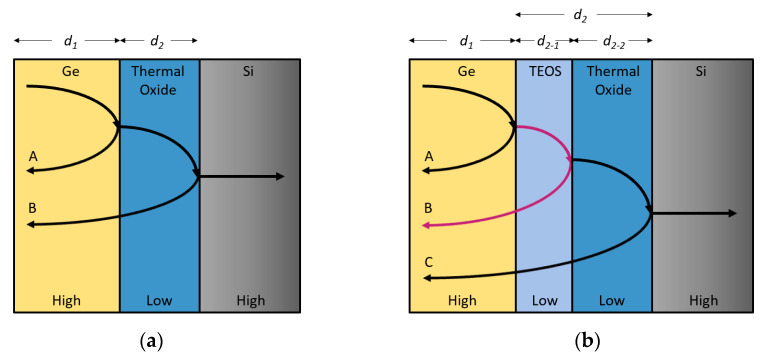
The schematic illustration of the principle of the dielectric mirror with high and low refractive index layers of (**a**) Ge/thermal-oxide/Si, and (**b**) Ge/TEOS/thermal-oxide/Si. Reflected waves A and B interfere constructively if the layer thicknesses *d_2_* is one-quarter of a wavelength within the layer.

Due to the high refractive contrast between Ge(n~4.2), SiO_2_(n~1.45), and Si(n~3.42), the light propagating in the Ge active layer can experience strong reflection at the Ge/insulator/Si interfaces, achieving better optical confinement in the GOI structure [18]. Due to the resonant cavity effect, the light intensity in the GOI active layer is higher than that of Ge on Si under the same light power irradiation, so the photocurrent of GOI PD is higher than that of Ge-on-Si PD. The schematic illustration of the principle of the dielectric mirror with high and low refractive index layers (Ge/Oxide/Si) is shown in Figure 7a. The thicknesses of Ge and SiO_2_ are *d*_1_ and *d*_2_. To enhance reflected light inside the Ge layer, A wave and B wave should interfere constructively; this requires the phase difference to be 2π. Thus, *d*_2_ needs to satisfy the following formula [38]:

*d*_2_ = *m*(*λ*/4*n*) (3)

in which *m* = 1, 3, 5… is an odd integer. λ is the free-space wavelength. *n* is the refractive index of SiO_2_. When *m* = 2, 4, 6…. is an even-integer, the A wave and B wave will interfere destructively. For wavelengths of 1550 nm, the calculated *d*_2_ for constructive interference could be 801 nm. The thickness of the SiO_2_ layer of GOI with TEOS is ~800 nm, which is very close to the calculated result of *d*_2_; therefore, it is more beneficial to form constructive interference in the Ge layer. Furthermore, the GOI with TEOS contained two types of oxide layers, TEOS and thermal oxide. The two-layer oxide structure with slightly different refractive indexes also provided a stronger optical resonant cavity effect for the detector, as shown in Figure 7b. That is, the GOI PD with TEOS had better optical confinement and stronger resonant cavity effect, resulting in its higher photo current than that of GOI PD with only a thermal oxide thickness of 523 nm. Table 2 shows the effect of different SiO_2_ thicknesses of GOI PDs on the light waves in the Ge layer. Thus, to obtain higher responsivity under 1550 nm and 1310 nm, the thickness of the oxide layer of GOI PDs should be accurately formed. In addition, the multi-layer structure can be used to realize the enhancement of the resonant cavity effect.

### 3.3. Spectral Response

**Figure 8:**
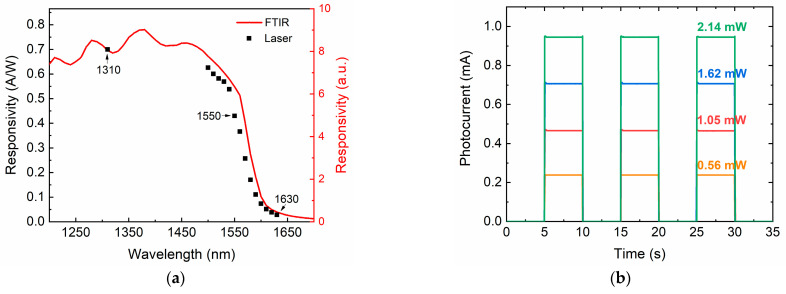
(**a**) The spectral response of GOI photodetectors between 1200 and 1700 nm, and (**b**) time-resolved photocurrent of the GOI photodetector at a wavelength of 1550 nm.

In order to investigate the broad spectral responsivity of the GOI detector, the spectral response of the photodetector was measured using a Nicolet 8700 Fourier-transform infrared spectrometer (FTIR) (Thermo Scientific, Waltham, MA, USA) with a KBr beam splitter and glow-bar source at room temperature. A commercial InGaAs photodetector was used to calibrate spectrum responsivity. Figure 8a shows the spectral response of the GOI PD without TEOS under zero-bias. The optical responsivities of 0.7, 0.43 and 0.028 A/W at −1 V obtained by laser under 1310 nm, 1550 and 1630 nm are also shown for comparison. The trend of spectral response is consistent with that measured by laser. Compared to the other Ge PDs reported previously [18,26,36], this GOI detector achieved high responsivity in a wide spectral range of 1200~1650 nm. The responsivity spectrum of GOI PD showed strong oscillation structures, indicating the effectiveness of the resonant cavity structure to enhance the responsivity. We strongly believe that the responsivity can be further improved: (i) by engineering the cavity length (the thickness of the Ge active layer and oxide layer); and (ii) by optimizing the device process. Moreover, the time-resolved photocurrent response of the GOI PD with a diameter of 80 μm at −1 V under different incident light powers at a wavelength of 1550 nm is shown in Figure 8b. The consistent and repeatable photocurrent responses are observed without noticeable degradation while the incident light is switched with 5 s period. This indicates that our Ge PDs have low defect density and good performance.

Therefore, we conclude that the GOI PDs are promising candidates for telecommunication applications with their extended photodetection range, enhanced optical responsivity, and complementary metal oxide semiconductor compatibility.

For comparison, an overview of the obtained results of the reported Ge-on-Si p-i-n photodetectors are listed in Table 3. To the best of our knowledge, our GOI p-i-n photodetectors proposed in this study exhibit the best comprehensive performance among the reported results. 

## 4. Conclusions

We have demonstrated a GOI substrate with a high-quality strained Ge layer. Optical devices (PDs) were prepared using a standard CMOS process on these substrates. The optical responsivity of the fabricated GOI p-i-n photodetectors with TEOS at 1550 nm and 1310 nm were 0.50 and 0.90 A/W at −1 V, corresponding to the external quantum efficiencies of 40% and 85.2%, respectively. The GOI p-i-n photodetector with both a thermal oxide and tetraethylorthosilicate (TEOS) layer showed the best optical performance due to its better optical confinement and resonant cavity effect. The GOI PDs without TEOS with a mesa diameter of 10 μm exhibited dark currents as low as 2.5 and 3.8 nA at −1 V and −2 V, and exhibited remarkable rectifying behavior with a high on/off current ratio near 10^8^ between 1 and −1 V. These high-performance GOI PDs with extended detection range to 1630 nm indicate that the GOI substrates and devices are ideal for telecommunications and Si-based monolithically integrated optoelectronics compatible with the CMOS process.

## 5. Patents

The patent of resonant cavity substrate structure for improving the responsivity of photodetector and its method has been submitted.

## Figures and Tables

**Table 1:** Dark current density comparison of PDs on three types of substrates.

Dark Current Density	Ge-on-Si PD	GOI PD with TEOS	GOI PD without TEOS
*J_bulk_* (mA/cm^2^)	2.50	2.02	1.79
*J_surf_* (μA/cm)	1.45	0.37	0.34

**Table 2:** The effect of different SiO_2_ thicknesses of GOI PDs on the light waves in the Ge layer.

GOI	Oxide Thickness		
*m* value	1	2	3
effect	Constructive	Destructive	Constructive
1550 nm	267 nm	534 nm	801 nm
1310 nm	226 nm	452 nm	678 nm

**Table 3:** Performance comparison for the top illuminated Ge p-i-n photodetectors.

Year	Active Area (μm^2^)	Dark Current at −1 V (nA)	Dark Current Density at −1 V (mA/cm^2^)	Responsivity at 1550 nm (A/W)	Responsivity at 1310 nm (A/W)	Quantum Efficiency (1550 nm/1310 nm)	Ref
2005	π × 2500	1728 at −2 V *	22 at −2 V	0.56 at −2 V	0.87 at −2 V	45%/82% *	[39]
2008	20 × 20	6.8 *	1.7	0.2 at −1 V	0.3 at −1 V	16%/28% *	[35]
2009	π × 25	102 *	130	0.05 at −2 V	0.2 at −2 V	4%/19% *	[40]
2010	50 × 50	990	40	0.13 at −1 V	——	10.4%/—	[10]
2013	π ×100	120 *	38.3	0.30 at −1 V	——	24%/—	[11]
2017	π × 36	5.2	4.6	0.27 at 0 V	0.59 at 0 V	21.6%/55.8%	[36]
2017	π × 900	1329 *	47	0.39 at −2 V	——	31.2%/— *	[27] GOI
2019	π × 25	4	5	0.12 at −1 V	——	10%/— *	[41]
2020	π × 56.25	450	255 *	0.31 at −1 V	0.52 at −1 V	24.8%/49.2%	[12]
2020	π × 15625	280 *	0.57	0.28 at −1 V	——	22.4%/— *	[26] GOI
This work	π × 25	6.4	8.1	0.32 at −1 V	0.54 at −1 V	25.6%/51.1%	Ge on Si
This work	π × 25	2.5	3.2	0.43 at −1 V	0.70 at −1 V	34.4%/66.3%	GOI WO-TEOS
This work	π × 25	2.7	3.4	0.50 at −1 V	0.90 at −1 V	40%/85.2%	GOI W-TEOS

The data have been extracted from the references as indicated. * Data calculated using the referenced material.

## Data Availability

The data presented in this study are available on request from the corresponding authors.
